# Rethinking the frequency code: a meta-analytic review of the role of acoustic body size in communicative phenomena

**DOI:** 10.1098/rstb.2020.0400

**Published:** 2021-12-20

**Authors:** Bodo Winter, Grace Eunhae Oh, Iris Hübscher, Kaori Idemaru, Lucien Brown, Pilar Prieto, Sven Grawunder

**Affiliations:** ^1^ Department of English Language and Linguistics, University of Birmingham, Birmingham, UK; ^2^ Department of English Language and Literature, Konkuk University, Seoul, Korea; ^3^ URPP Language and Space, University of Zurich, Zurich, Switzerland; ^4^ Department of East Asian Languages and Literatures, University of Oregon, Eugene, OR, USA; ^5^ School of Languages, Literatures, Cultures and Linguistics, Monash University, Melbourne, Australia; ^6^ ICREA, Institució Catalana de Recerca i Estudis Avançats, Barcelona, Spain; ^7^ Departament de Traducció i Ciències del Llenguatge, Universitat Pompeu Fabra, Barcelona, Spain; ^8^ Department of Empirical Linguistics, University of Frankfurt, Frankfurt, Germany; ^9^ Department of Human Behavior, Ecology and Culture, Max Planck Institute for Evolutionary Anthropology, Leipzig, Germany

**Keywords:** pitch, politeness, sound symbolism, prosody, intonation, vocal dominance

## Abstract

The widely cited frequency code hypothesis attempts to explain a diverse range of communicative phenomena through the acoustic projection of body size. The set of phenomena includes size sound symbolism (using /i/ to signal smallness in words such as *teeny*), intonational phonology (using rising contours to signal questions) and the indexing of social relations via vocal modulation, such as lowering one's voice pitch to signal dominance. Among other things, the frequency code is commonly interpreted to suggest that polite speech should be universally signalled via high pitch owing to the association of high pitch with small size and submissiveness. We present a cross-cultural meta-analysis of polite speech of 101 speakers from seven different languages. While we find evidence for cross-cultural variation, voice pitch is on average lower when speakers speak politely, contrary to what the frequency code predicts. We interpret our findings in the light of the fact that pitch has a multiplicity of possible communicative meanings. Cultural and contextual variation determines which specific meanings become manifest in a specific interactional context. We use the evidence from our meta-analysis to propose an updated view of the frequency code hypothesis that is based on the existence of many-to-many mappings between speech acoustics and communicative interpretations.

This article is part of the theme issue ‘Voice modulation: from origin and mechanism to social impact (Part I)’.

## 1. Introduction

Pitch serves numerous functions in human communication. In all languages, pitch is used for intonation. For example, English speakers can use pitch to signal new information or contrast (e.g. *It was THIS*↑ *one*) or to communicate the difference between questions and statements (e.g. *You went to the movies?*↑ versus *You went to the movies.*↓) [1–4]. In many languages, pitch is also used to mark lexical contrasts [5,6], such as in Mandarin Chinese, where the syllable *ma* means ‘mother’, ‘hemp’, ‘horse’ or ‘scold’ depending on the specific tone used. Beyond these linguistic functions, pitch also communicates a range of affective, attitudinal and social meanings, including emotions such as anger and joy [7,8], as well as attractiveness, dominance, masculinity or strength [9–13].

The frequency code hypothesis proposed by Ohala [14] seeks to relate a large number of these diverse communicative functions to the acoustic projection of body size. Across the animal kingdom, larger bodies often produce vocalizations with lower frequencies [15–18], and human listeners perceptually associate low pitch and low resonance frequencies with larger bodies [19,20]. This link between acoustics and body size is proposed to motivate a diverse range of communicative uses of pitch, including the following three related classes of facts: (i) social indexing, such as using vocal modulation to signal dominance and deference; (ii) size sound symbolism, such as using pitch to iconically depict size differences; and (iii) proposed universal tendencies in intonation phonology, in particular the use of rising pitch contours to signal questions [21,22]. The frequency code hypothesis is a highly synergetic proposal, suggesting that pitch is associated with a large and diverse ‘package of evolutionary meanings' [23, p. 81]. It is, moreover, a very bold proposal, suggesting that a number of different classes of cultural facts have a singular shared biological motivation.

We first provide a detailed review of the original frequency code hypothesis in the light of new evidence (§2). Then we test one of the frequency code's predictions—that politeness should be associated with high pitch—in a meta-analysis of existing studies from seven languages (§3). This new evidence leads us to propose a more multidimensional account of the role of body size in human vocal communication (§4).

## 2. The frequency code

### (a) Background

When considering size-related acoustics in humans and other animals, it is important to distinguish between fundamental frequency (*F*0), determined by the speed of vocal fold vibration and resonance frequencies, determined by the size and shape of the resonator. This difference can be characterized in terms of ‘source-filter theory’ [24], a two-stage model of vocal production where the glottal wave generated by the vocal folds in the larynx (the ‘source’) is subsequently manipulated in its spectral characteristics by the shape and size of the supralaryngeal vocal tract (the ‘filter’). Humans have vocal control over both source and filter: they can modulate the pitch by vibrating their vocal folds faster (source), and they can create different vowels and consonants by changing the position of the tongue (filter). The resonance frequencies of the vocal tract, called ‘formants’, are the primary determinant of vowel quality, such as the difference between /i/ and /a/. Throughout this paper, we distinguish between *F*0 and formant frequencies, but we refer to ‘vocalization frequency’ when the distinction between the two is irrelevant, for example, because the two lead to similar effects in perception [20].

All else being equal, larger animals have lower vocalization frequencies [25]. This is the case with fundamental frequency, which is correlated with body size when making comparisons across different animal species, as has been established for birds [26], frogs [17] and mammals [15,27], among others. Vocalization frequencies (formant frequencies or *F*0) may also reflect body size within a given species, as has been shown for red deer and fallow deer [28,29], macaques [16], koalas [27], alligators [30] and others.

As is the case with many other mammals (e.g. [31,32]), human vocalizations are characterized by sexual dimorphism: the fundamental frequency of female speakers is on average around 70% higher than that of male speakers [33]. However, the range of *F*0 values overlaps between male and female speakers and is subject to culturally and individually variable vocal modulation, such as when speakers actively change pitch to produce a range of gendered meanings (e.g. [34–36]). Despite this cultural and individual variation, listeners are highly sensitive to the distinction between male and female voices, a skill that arises early but continues to develop throughout one's lifetime [37]. Evidence from auditory Stroop tasks with male and female voices shows that people automatically process the speaker's gender even when this information is task irrelevant [38]. This automaticity of paying attention to vocal gender cues arises relatively early in development [39]. While gender is complex and goes much beyond biological sex differences, this literature shows that vocal sex differences are highly salient to listeners. This is important for the discussion of acoustic body size in humans: regardless of whether there actually is or is not a correlation between *F*0 and body size within sexes, people experience the association between *F*0 and body size *across* sexes. In addition to sex differences, there is a clear correlation between body size and acoustics across age, with small infants having much higher fundamental frequencies and resonance frequencies than adults who are also larger [40,41].

*F*0 does not reliably track differences in body size among adult speakers within sexes [42–44], although formant frequencies do [42]. To some extent, what is the *veridical* acoustic correlate of body size does not matter when it comes to communicative interpretations of size-related acoustics: regardless of the fact that *F*0 is not a reliable cue to an adult speaker's body size, people perceptually associate lower-pitched voices with larger speakers [20]. Similarly, when humans exaggerate their speech to give the impression of increased body size, they actively lower their *F*0 [45]. Three-month-old infants already associate low *F*0 and low formants with larger bodies [19], suggesting that the sensitivity to acoustic body size arises early.

### (b) The frequency code hypothesis

Ohala's frequency code hypothesis extends these facts about the acoustic correlates of body size into the realm of various social and linguistic phenomena [14]. The frequency code is a bold and synthetic proposal, bringing together a number of seemingly disparate communicative facts by proposing a biological motivation grounded in body size differences between animals. At its most general level, the frequency code proposes that a number of different social and communicative interpretations of pitch and vowel quality derive from whether something sounds ‘small’ (=high frequency sounds) or ‘large’ (=low frequency sounds), which is also why the frequency code has been called a ‘size code’ [23,46]. Crucially, being grounded in biology does not entail that acoustics are tied to the body in a static manner. As pointed out by Gussenhoven [46], ‘communication by means of the codes does not require that these physiological conditions are actually created. It is enough to create the effects. That is, the effects are not automatic, but have been brought under vocal control’ [46, p. 48].

In the following, we review new empirical evidence relevant to the frequency code. With respect to the social dimension, Ohala [14, p. 327] proposed that social messages such as ‘deference, politeness, submission, lack of confidence, are signaled by high and/or rising *F*0 whereas assertiveness, authority, aggression, confidence, threat are conveyed by low/or falling *F*0’. The idea that low *F*0 and lower formant frequencies are associated with the vocal expression of dominance has been extensively confirmed, both in production and in perception [9,10,12]. The frequency code would see the vocal signalling of dominance as being connected to the acoustic projection of body size. However, size is not the only predictor of physical dominance [47], suggesting that other factors may be associated with the perceived connection between low *F*0 and dominance. For example, low *F*0 is also independently associated with high testosterone levels [48–50].

An issue with Ohala's discussion of social messages is that several of the social terms he uses are left undefined. This is especially problematic with respect to such socially and culturally variegated phenomena as politeness, a topic for which there has been extensive debate about definitional matters (e.g. [51,52]). Politeness actually has many different manifestations (e.g. [51,53–55]), and it is not clear which one of these Ohala's claims relates to. Although Ohala [14] does not directly specify how body size is linked to politeness and deference (nor define how he understands these terms), it appears that this is based on the idea that being polite or deferential is speaking in a subdominant or submissive manner. Indeed, universal theories of politeness that appeared around the same time saw some modes of politeness as working along these lines. Notably, Brown & Levinson [54] describe one mode of politeness (what they call ‘negative politeness’) in terms of using strategies that make the speaker appear weaker and less likely to threaten or impinge on the interlocutor. In addition, they saw power differences as being a factor that resulted in increased levels of politeness. At the time the frequency code was proposed, most of the available evidence appeared to confirm that politeness was indeed associated with the high pitch in languages such as English [56,57], Japanese [56] and Tzeltal [54], consistent with the idea that the role of high pitch in politeness may stem from signalling submissiveness by acoustically projecting ‘smallness’. However, as we will discuss below, newer evidence calls into question the claim that high pitch is universally associated with politeness, or at least certain forms of it.

In addition to these social meanings, Ohala explicitly links the frequency code to the concept of ‘sound symbolism’. Specifically, he suggested that ‘words denoting or connoting the concepts small or smallness tend to exhibit a disproportionate incidence of vowels and/or consonants characterized by high acoustic frequency’ [14, p. 335]. In English, adjectives for small size (*tiny, wee, itsy-bitsy, mini, little, meagre, petite*, etc.) are more likely to contain the high frequency sounds /i/, /ɪ/ and /t/, as opposed to adjectives for large size (*large, great, vast, whopping, gargantuan, colossal*, etc.), which are more likely to contain /a/ [58]. Cross-linguistic evidence shows that indeed, translation equivalents of the words *small* as opposed to *large* are statistically much more likely to contain the high-front vowel /i/ than low-back vowels such as /a/ and /o/ [59–62]. Size sound symbolism also matters in naming: shorter and lighter American baseball players are more likely to receive nicknames with high vowels than taller and heavier baseball players [63]. Experimental evidence furthermore shows that English speakers associate novel made-up words and product names such as *mil* with smaller concepts than words with low vowels such as *mal* [64–68]. Similar cross-modal associations between speech sounds and semantic size have been experimentally established for a multitude of different languages [69–74], all of which corroborates the sound symbolic component of the frequency code: speakers do indeed perceptually map the vocal frequencies of speech sounds onto semantic size, and this cross-modal connection is reflected in the phonological patterns of size terms across languages.

Size sound symbolism has also been found for tone languages. Already in 1927, Westermann [75] made the observation that in the African languages Ewe, Twi and Nupe, words for small, narrow, light and quick concepts tend to be expressed by words with high lexical tones, in contrast with words for large, broad, heavy and slow concepts, which are associated with low lexical tones. That is, even though lexical tone is generally thought to be a primarily arbitrary feature of a language that merely serves to make contrasts between words, the association of tones with words can also be directly motivated by the connection between acoustics and body size. The observation that high lexical tones are associated with smallness has since then been made for other African languages, including Yag Dii [76] and Bini [77]. However, we are not aware of any typological work to demonstrate that small meanings are statistically more likely to be encoded with a high tone across a large sample of genealogically diverse languages.

The final extension of the frequency code is into the domain of intonation and prosody. It has been proposed that signalling question intonation with rising pitch contours and statements with falling contours is a universal tendency across languages [21,22,78,79]. Ohala [14, p. 331] offers an explanation for this universal tendency that relates to acoustically projected body size via associated dominance (low-pitched = larger and more dominant; high-pitched = smaller and more submissive). Specifically, he argues that ‘one need only allow that the person asking a question is, from an informational standpoint, in need of the goodwill and co-operation of the receiver. The questioner, as it were, is appealing to the addressee for help’. By contrast, Ohala says that ‘The person making a statement is self-sufficient’. There are multiple interpretations of this proposed link between question universals and size-related meanings. One idea reflected in the above characterization appears to be that a question asker is ‘informationally submissive’, somebody who literally does not know something, expressing a lack of confidence intonationally.^1^ A statement, on the other hand, is informationally ‘dominant’—things are known and can be said with authority. In addition, a question is potentially imposing, i.e. a question has the potential to be a face-threatening act [54]. This may thus require politeness strategies, such as making oneself appear less imposing by virtue of signalling submissiveness. However, it has to be pointed out that Ohala's explanation of the link between body size, dominance and question intonation is fairly indirect, requiring a lot of linking assumptions. We return to this point in our Discussion section.

### (c) Zooming in on politeness, and the need for more research

As discussed above, there is much empirical evidence that can be seen as confirming aspects of the original frequency code, such as several new experimental studies finding low pitch to be associated with dominance in production and perception [10,12], or new typological studies showing that high-front vowels are indeed associated with the concept of smallness across the world's languages [59]. When Ohala originally proposed that deference and politeness ‘are signaled by high and/or rising *F*0’, there was very little empirical evidence available for this claim. Now that multiple studies have investigated politeness in a number of languages, it is important to take stock of the available cross-linguistic evidence.

The study of vocal modulation to achieve politeness effects is still in its infancy. This is in part because traditionally politeness research emphasized verbal markers of politeness, such as politeness expressions (e.g. *Please, Thank you*), indirect speech acts (e.g. *Can you pass me the salt?* as opposed to *Pass me the salt*) or various forms of honorifics, as can be found in languages such as Korean and Japanese, where verbs are inflected depending on the social connection with the interlocutor [80–82]. Recently, a number of studies in politeness research have started to go beyond the verbal domain and explored to what extent politeness meanings are signalled non-verbally via speech and gesture [83–90].

A number of studies on non-verbal politeness appear to confirm the notion that deference and politeness are associated with high pitch, as predicted by the frequency code. For example, it has been shown that Japanese speakers, particularly females, use higher pitch when using politeness formulae [56] or when talking to customers and clients [91]. In addition, it has been found that Japanese speakers use higher pitch on the final vowel of the sentence when addressing someone of higher social standing [36,92]. Similarly, Canadian English speakers use higher pitch in indirect polite requests (*Can you lend me a nickel, please?*) and lower pitch in direct requests (*Lend me a nickel!*) [93]. In Mexican Spanish, speakers favour high initial and a high final boundary tone in polite requests [94].

Table 1 shows an overview of production and perception studies that are relevant to the status of politeness in the frequency code hypothesis. The table includes studies that looked at different facets of politeness, and also closely related meanings such as formality. We only included studies that directly measure *F*0 acoustically (production), or directly manipulated *F*0 (perception). Studies that make descriptive observations without directly measuring or manipulating *F*0 (e.g. [54,86]) are excluded. In addition, we excluded studies which show that acoustics matter to politeness perception if these did not directly investigate the effects of *F*0 [108,109]. We also excluded studies that measured other acoustic features if they did not also include *F*0 measurements (e.g. [110] on Japanese voice quality in polite speech) or did not report *F*0 measurements (e.g. [111]).

**Table 1.** Empirical studies on politeness-related phenomena that directly measure F0 or manipulate it (perception experiment); studies marked by an asterisk are included in our meta-analysis.

author/year	study type	participant sample	results
Loveday 1981 [56]	production	5 Japanese speakers (2 female)	Japanese female speakers used artificially high pitch in formulaic politeness expressions
		5 English speakers (2 female)	
Ofuka *et al.* 2000 [92]	production/perception	6 Japanese speakers (all male)	variable pitch results in production; final rises interpreted to be more polite; medium levels of speech rate more polite
		20 Japanese listeners (8 female)	
Ohara 2001 [36]	production	5 Japanese speakers (all female)	higher pitch in polite speech
Nadeu & Prieto 2001 [83]	perception	20 Catalan listeners (13 female)	increased pitch led to increased politeness judgements only in the presence of a happy face (experiment 2)
Goodwin *et al*. 2002 [95]	production	10 Spanish/English speakers (all female)	stylized high pitch contours for disagreement
Chen *et al.* 2004 [96]	perception	53 Dutch and 29 British English listeners (gender not specified)	both languages interpreted higher pitch registers to be more ‘friendly’
Tsuji 2004 [91]	production	8 English speakers	high pitch used to mark friendliness in English, deference in Japanese; Japanese speakers use high pitch in service speech
		8 Japanese speakers (4 female each)	
Shin 2005 [97]	production	6 German; 6 American English	higher pitch when speaking to friend as opposed to professor
		6 Korean speakers (3 female each)	
Stadler 2006 [98]	production (corpus)	220 utterances from televised New Zealand and German panel discussions	disagreement produced with high pitch
Orozco 2008 [94]	production	12 Mexican Spanish speakers (6 female)	polite requests involved high final boundary tone and high initial tone
Winter & Grawunder 2012* [87]	production	16 Korean speakers (9 female)	lower pitch in polite speech, also lower shimmer and jitter, higher H1–H2, slower, quieter
Devís & Cantero 2014 [99]	production (corpus)	160 Catalan speakers (corpus)	politeness markers involve final rises
Grawunder *et al.* 2014* [100]	production	13 German speakers (11 female)	lower pitch, higher harmonics-to-noise ratio (HNR), higher pitch range, lower intensity in polite speech
		18 Austrian speakers (8 female)	
Hübscher *et al.* 2017* [101]	production	20 Catalan speakers (all female)	lower pitch in polite speech; also slower speech rate; less intensity, shimmer, jitter; increase in H1–H2
Chikulaeva & D'Imperio 2018 [102]	production	11 Russian speakers (all female)	all pitch accents with the exception of downstepped H+!H* showed higher *F*0 for polite as opposed to impolite speech
Caballero *et al.* 2018 [93]	perception	48 Canadian English listeners (24 female)	compared to rude requests, polite ones were high-pitched, slower
Idemaru *et al.* 2019* [103]	production	20 Japanese speakers (12 female)	no reliable difference in pitch; polite speech was quieter and had higher HNR, lower jitter, higher H1–H2
Sherr-Ziarko 2019 [104]	production	10 Japanese speakers (5 female)	lower pitch in polite speech; also quieter, slower
Idemaru *et al.* 2020 [105]	perception	63 Korean speakers (32 female)	no reliable perceptual difference resulting from pitch, but quiet speech interpreted as more polite
Gucek & Le Gac 2019 [106]	production	9 Porteño Spanish speakers	lower pitch in polite speech
Oh & Cui 2020* [107]	production	8 Chinese speakers (4 female)	lower pitch, quieter, higher H1–H2, higher HNR, slower in polite speech

Table 1 shows that there are a number of studies that can be thought of as contradicting the frequency code. These studies either find that speakers actively lower their voice pitch in a polite condition as opposed to a comparison condition [87,97,100,101,104,106,107] or they find no consistent pitch difference [103,105]. A look at table 1 makes it apparent that the correlates of polite speech are quite variegated, sometimes even when it comes to different studies of the same language. For example, for Japanese, there is evidence that is consistent with the idea that high pitch signals politeness [36,56,92], as well as studies that found a lowering of pitch [104], as well as studies that found no reliable differences [103]. However, one issue that makes it hard to establish any overarching cross-linguistic tendencies is that the studies used different tasks, as well as different definitions of politeness. The comparison condition that is used to contrast with the polite condition also differs across these studies. For example, Caballero *et al*. compared polite requests to rude requests, but other studies compared polite to ‘neutral’ language [98] or to ‘informal’ language [87]. The diversity of studies motivates the need to have an integrated analysis of those studies that are more directly comparable.

## 3. Meta-analysis of politeness data

In this section, we follow up on the idea that the frequency code predicts an association of high pitch with politeness. We use the evidence from politeness to demonstrate the need to qualify and extend the frequency code. Crucially, the evidence we present only addresses one component of the overall proposal (and potentially only one facet or manifestation of it), but it demonstrates important conceptual issues that help in revising the account.

### (a) Task

The present paper synthesizes the data from a number of existing studies on different languages, all of which involved the current authors. We specifically focused on studies that followed up on Winter & Grawunder's [87] methodology, of which there are several by now. This allows us to look at the role of the pitch for one homogeneous task, thereby facilitating evidence synthesis.

The task is a spoken version of what is called the Discourse Completion Task [112], which involves responding to specific discourse contexts in an appropriate manner, such as imagining to ask a professor for help. Each participant was asked to render a number of different utterances for distinct scenarios (‘unique items’ in table 2) to an imagined superior or to an imagined friend or status-equal peer. The full list of scenarios/items is available in the following Open Science Framework repository: https://osf.io/amw7u/.

**Table 2.** Overview of participant and item sample from the different studies.

language/study	*n* of participants	age range	*n* unique items
Korean [87]	16 (9 female)	21–31	5
Japanese [103]	20 (12 female)	19–21	6
Chinese [107]	8 (4 female)	17–20	10
Catalan [101]	20 (20 female)	18–29	6
Austrian German [100]	18 (8 female)	19–29	8
German [100]	13 (11 female)	18–27	8
Russian (unpublished)	6 (5 female)	18–23	7

A Korean example of a specific set of responses from one participant in one condition (polite and non-polite) is given below. The situation involves telling a driver that they have missed a turn.

(a) Polite condition (addressee = superior)
  1. *a, pwucangnim a, ssup!*
  ‘oh, chief, oh, ssup [inhalation]’
  2. *ceyka alki-lo-nun ceccok kil-lo kaya toy-nun ke kath-untey, ssup*
  ‘as far as I know, I think we should have turned down that road, ssup [inhalation]’
  3. *cinachin ke katha-yo*
  ‘it looks like we've gone past it’
  4. *ssup*
  ssup [inhalation]
  5. *ceki han 30mithe kakac-ko cha tolli-si-ko*
  ‘go about another 30 meters and turn around’
  6. *ceccok-ulo ka-si-eya toyl ke katha-yo*
  ‘I think we have to go that way’.
(b) Non-polite condition (addressee = friend)
  1. *ya cinass-canha*
  ‘hey, we've gone past it’
  2. *a ppalli kkekke*
  ‘turn quickly’
  3. *kkekke*
  ‘turn’
  4. *cekiya*
  ‘that way’
  5. *ceki*
  ‘that way’

This example focuses on a particular type of politeness expression, specifically the modulation of speech depending on the hierarchical (power) relationship with the interlocutor. We can see in the example above that the difference in the relationship already results in a number of differences on the verbal level. The version addressed to the status superior is longer, uses more indirect and uncertain expressions, includes honorific morphemes (such as –*yo* in lines 3 and 6 and –*si*– in lines 5 and 6), and features audible breath intakes that have a hissing quality.

The appropriate modulation of speech according to the relationship with the interlocutor is crucial to politeness theory [54], and has been referred to in research on politeness by terms such as ‘discernment politeness' [113] and ‘bivalent politeness’ [114]. In this paper, we refer to the deferential and more formal speech addressed to a superior as ‘polite’, and the casual and more informal speech addressed to a friend as ‘non-polite’. These labels are used for convenience and should not be understood as interpretations of how these levels of speech may be understood in context: ‘non-polite’ speech may be perfectly appropriate for addressing a friend or status-equal peer, and using ‘polite’ speech does not guarantee a polite interpretation. In fact, it has been shown that politeness markers can sometimes be used to achieve the opposite effect (e.g. [115]). Ultimately, politeness does not reside in a particular style of speech, but in how that style is used and interpreted in context [55]. The labels ‘polite’ versus ‘non-polite’ are a convenient shorthand for expressing the relative difference that matters to this task: speech oriented towards an older and socially more distant status superior, as opposed to speech oriented towards a relatively younger and relatively more intimate status-equal.

### (b) Participants

Table 2 gives an overview of the participant sample, which includes data from seven languages spanning four distinct language families (Indo-European, Sino-Tibetan as well as Japanese and Korean). On the Indo-European side, the data span three distinct subgroups within the family (Romance: Catalan; Germanic: German; Slavic: Russian).

### (c) Acoustic analysis

To ensure consistent measurement, we did not rely on the reported *F*0 means from the existing studies but instead extracted *F*0 from the raw acoustic data of each of the studies, using the Praat [116] autocorrelation algorithm with the following settings: 10 ms time-step Gaussian window, 75 Hz pitch floor, 500 Hz ceiling, 15 candidates, 0.035 silence threshold, 0.6 voicing threshold, 0.01 octave cost, 0.35 octave-jump cost and 0.14 voiced unvoiced cost. These pitch settings were motivated based on extensive hand-checking of all items to assess the presence of pitch tracking errors. A few isolated responses reached all the way up to 500 Hz, which is why we chose this specific pitch range. We filtered out artefacts beyond the outer 0.02 quantiles.

Our primary dependent measure is a set of *F*0 measurements that are based on the median of the entire set of utterances spanning each response (results reported below also hold for means and are even stronger in that case). Our focus on *average F*0 values (medians), rather than maximum *F*0 or *F*0 span, is motivated for several reasons: first, this is the primary measure discussed in other studies relating to the frequency code, such as studies of vocal masculinity (e.g. [12]). Second, the average *F*0 is the primary measure that is consistently discussed across all the studies that we perform a meta-analysis of. Third, the average *F*0 more directly corresponds to the idea of acoustically projected body size (which should characterize pitch at a global level), in contrast with the maximum and minimum values. Similarly, pitch span, although theoretically interesting, is not of primary concern here as this measurement has been argued to correspond to a different biological code, the effort code [23,96]. Obviously, our analysis does, therefore, not account for the fact that more specific aspects of the intonation contour may also signal body size and/or politeness-related meanings, which is the focus of other studies (e.g. [92,96]).

Given that participants were free to respond in any way they deemed fit, it is important to emphasize that the responses were not lexically equivalent (as shown in example (1) above). This means that the acoustics will also be driven by the specific lexical choices made by participants, which has the potential of introducing confounds, i.e. it is possible that more words with higher intrinsic pitch were uttered in the polite condition. It is, however, quite unlikely that our results would be driven by these confounding factors because the different languages we investigate have non-overlapping lexical strategies for marking politeness. Moreover, given that there was considerable variation in how participants approached the task (with responses being largely non-overlapping in the choice of lexical material), it is quite unlikely that speakers of multiple genetically unrelated languages would select lexical material that happens to involve phonemes with consistently higher/lower intrinsic pitch. Finally, the alternative methodological option of keeping responses lexically uniform would make the task even more artificial and potentially diminish any acoustic differences, given that read speech is less expressive. To the extent that the datasets that are part of our meta-analysis have more variation than what is usually present in phonetic research on speech production or intonation, any consistent *F*0 difference we observe is even more compelling and makes the results more generalizable (cf. [117]). Finally, it should be emphasized that similar studies, such as those on vocal masculinity [10], have also analysed overall acoustic differences for variable utterances and found consistent *F*0 differences.

### (d) Statistical analysis

All statistical analyses were conducted with the R programming language v. 4.0.2 [118] and the tidyverse package 1.3.0 [119]. The main analysis, a Bayesian mixed effects regression, was implemented with the package brms 2.13.3 [120]. In this model, the median *F*0 across the whole target utterance of each trial (for each speaker each unique item in each of the two conditions) was the dependent variable. As a fixed effects predictor, we include gender and condition (polite versus non-polite). As random effects, we include by-participant and by-item varying intercepts, as well as by-participant and by-item varying slopes for the condition effect. In addition, we included language as a random effect (by-language varying intercepts and by-language varying condition slopes), similar to how language family is a random effect in a lot of typological research [121,122]. Given the low number of languages per language family (including two isolates: Japanese and Korean), it is impossible to fit a language family random effect in this case.

All data and code are available under the following repository: https://osf.io/amw7u/.

### (e) Results

**Figure 1.**
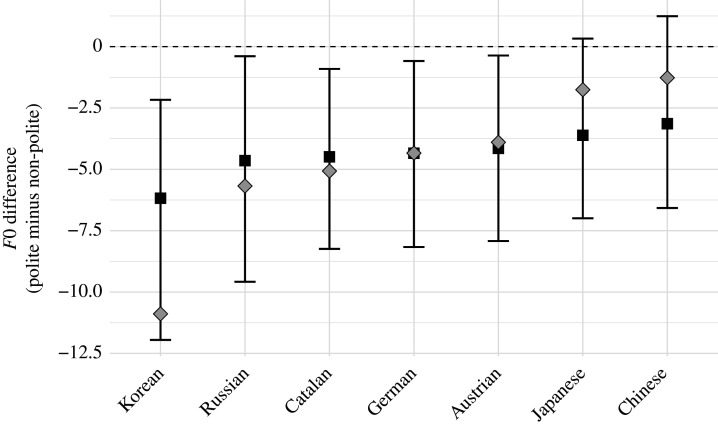
Posterior means (squares) with 95% credible intervals from our Bayesian mixed effects regression analysis; descriptive averages are superimposed grey diamond shapes; the individual observations are *F*0 values for polite and non-polite trials (medians over all utterances in response to a single discourse prompt).

Our results show that at least for our task, the pitch is either lowered (Korean, Russian, Catalan, German, Austrian German) or does not differ between the two politeness conditions (Japanese and Chinese); figure 1. Specifically, we found that polite speech was on average 4.3 Hz lower, with a 95% Bayesian credible interval ranging from –7.4 Hz to −1.1 Hz. The posterior probability of the polite speech being *higher* in pitch ($\beta_{\mathrm{diff}}>0$) was very low, *p* = 0.007.

It is also worth highlighting that this picture was fairly consistent across speakers. Across the sample, 75% of all speakers lowered their voice pitch in the polite condition (average across all items). This pattern was most pronounced for Korean speakers (94%), followed by Russian (83%), Catalan (80%), Japanese (70%), Austrian (72%), German (69%) and Chinese (38%) speakers. In the next section, we discuss the implications of these results for the frequency code hypothesis.

## 4. Rethinking the frequency code

In summary, our results show that at least for this particular task, the predictions of the frequency code with respect to politeness are not confirmed. It is worth mentioning that the particular type of politeness involved in this task should be the kind of politeness that is most amenable to showing results in line with the frequency code, since the key manipulation in the task is speaking to a superior, which could be taken to require a submissive speech style. Despite this, we found that polite speech in response to an (imagined) superior was consistently *lower* in pitch than speech to an (imagined) intimate friend or same-aged peer. In this section, we consider potential explanations for this finding, and we use this finding as a springboard to propose revisions of the frequency code hypothesis.

### (a) Pluripotentiality of pitch and prosodic mitigation

While Ohala himself explicitly referenced the idea that pitch can have multiple different meanings [14, p. 98], we believe that the large meaning potential of pitch is not often appreciated enough in interpreting research findings on vocal modulation, especially those findings that stem from experimental research that focuses on just one key manipulation. It is clear that pitch is what we would like to call *pluripotential*, having multiple different meanings that depend on numerous contextual factors. For example, Chen *et al*. [96] demonstrated that the same prosodic manipulation gives rise to different interpretations even among two closely related speaking communities (British English and Dutch).

The contextual factors that influence the interpretation of pitch can be fairly global (such as different cultural interpretations of high pitch within a speaking community at large) or very local, such as the particular situated context in which an interaction takes place. Another contextual factor is the presence or absence of other acoustic or gestural cues that are used alongside the vocal modulation of pitch. Polite speech is not just characterized by pitch alone but by numerous different phonetic parameters, e.g. we have found that lowered pitch also goes along with decreased loudness, decreased speech rate and clearer voice quality [87,101,103]. Depending on which of these cues are present in an utterance, the interpretation of pitch can change drastically. For example, a low-pitched utterance accompanied by clear speech has a fundamentally different interpretation from a low-pitched utterance accompanied by creaky glottal fold vibration that has a growling quality. A direct demonstration of how context modulates the politeness-related meaning of pitch within a particular language (Catalan) is given by Nadeu & Prieto [83], who showed that utterances with artificially increased pitch span led to decreased politeness judgements unless accompanied by a happy face, which completely reversed the effect of pitch span. This is an example of how a concomitant visual cue completely reverses the politeness-related meaning of pitch, which is a proof-of-concept demonstration of how pitch meanings are altered by context.

When it comes to the social meanings of pitch, the issue then is that high pitch alone (in contrast with low pitch) cannot only signal submissiveness [9,10,12,13], but also disagreement [95,98], certain forms of anger [123], and intense negative emotions such as disgust, anxiety and shame [7,8]. Thus, the same unidimensional acoustic cue—pitch—signals a whole range of different meanings, many of which could be seen as being incompatible with politeness. Without context, the meaning of pitch is underspecified. It is only within a given context and in accompaniment with other cues that the particular communicative interpretation of pitch becomes constrained.

It is important to remember that each experiment on the vocal signalling of social and affective meanings artificially constrains the pluripotentiality of pitch, thus either explicitly or implicitly taking a more unidimensional perspective. An example of this is a study conducted by Puts *et al*. [10], in which a competitive scenario was used to elicit vocal expressions of dominance and in which a group of independent listeners had to judge the vocalizations for dominance. The nature of both the production and the perception task constrains the range of possible interpretations that pitch can have. The isolation of a particular variable of interest is of course the hallmark of any good experiment, but it also means that the multidimensionality of the social and affective meanings of pitch may be underestimated vis-à-vis how pitch is deployed in situated interactions. Specifically, in any one naturalistic communicative scenario outside of the laboratory, multiple social and affective factors come into play simultaneously (power, distance, intimacy, emotional states, personas that speakers want to display etc.), as do additional phonetic and gestural cues.

The tasks that are part of our meta-analysis were all focused on just one dimension of politeness, specifically, the formal speech that is required when speaking with older, less intimate and more powerful interlocutors. More methodological diversity, including observational studies that look at actual face-to-face interactions, are needed in order to shed light on the variegated meanings of the pitch. It is also possible that our task, given its rather artificial nature reliant on initiating *imagined* interactions, would show smaller effect sizes than can be observed in more naturalistic settings, where politeness may play a more significant functional role, e.g. when there is a social cost for not being perceived as polite. Given these limitations inherent in our task, the consistency of our results is compelling.

However, it still remains to be explained why we have found consistently *lower* pitch in our task. In the context of addressing a status superior, far from being connected to dominance (as the frequency code would claim), we have claimed elsewhere [87,101] that lower pitch is connected to a polite and deferential way of talking via associations with a more formal, ‘damped down’ and composed mode of delivery [87], what we have also called ‘prosodic mitigation’ [101]. This politeness-as-prosodic-mitigation account is consistent with a number of different empirical findings: several of the other acoustic parameters investigated fit the interpretation of mitigated speech (e.g. quieter, slowed down, less variable in pitch and intensity). Beyond speech, prosodic mitigation is compatible with the observation that in formal polite interactions with status superiors, Korean speakers gesture less and assume more constrained and less mobile body positions [88]. Similar results have also been found for facial and overall non-verbal expressivity among Catalan speakers [124].

This is not to say, however, that prosodic mitigation is the only means of conveying politeness acoustically. On the contrary, previous studies show that in some contexts and some cultures using higher pitch is also available for certain politeness-related meanings, as shown in results supporting the frequency code [56,93,94]. Owing to its potential to sound ‘smaller’ and therefore more submissive, speakers may employ higher pitch to produce a demure and pliant demeanour, which may be polite in some contexts. The higher pitch has also been found to be associated with liveliness and friendliness [83,91], which may explain why previous studies that focus on politeness routines in service industry interactions (where demureness and/or friendliness may be valued highly) tend to show preferences for high pitch [56,91]. Politeness-related meanings are complex and interactionally variable, and are achieved via complex associations between vocal qualities and social meanings. The available evidence allows us to rule out a simple one-to-one mapping of the pitch to communicative interpretation.

In the following sections, we extend the idea of pluripotentiality to the other dimensions of the frequency code, including sound symbolism (§4.2) and intonational phonology (§4.3).

### (b) Pluripotentiality and sound symbolism

In the last section, we argued that pitch has multiple different meanings that are determined by context. This is actually even more clearly demonstrated in the domain of sound symbolism, which provides direct evidence for the same idea. What we call ‘pluripotentiality’ has been discussed in the sound symbolism literature as ‘plurisignificance’ [125] (see also [126,127]). For example, when participants are asked to match pure tones to objects of varying sizes and shapes, high pitch is matched not only to small objects, but also to triangular as opposed to round shapes [128]. People also reliably associate high pitch and high-front vowels with brighter stimuli than low pitch and low-back vowels [129–132]. In a review of the experimental literature on sound symbolism, Lockwood & Dingemanse [133] show that differences in vowel quality are associated with a large number of perceptual dimensions, including size, shape, brightness, speed, colour and taste (see also [134]). Miall [135, p. 60] says that ‘while phonemes have no intrinsic meaning—/i/ is not invariably small or bright—they possess a potential meaning capable of realization when a contrast is in question’ (see also [136–138]). How local context disambiguates the ‘potential’ iconic meanings of each sound was directly demonstrated by Winter *et al*. [139], who used the same experimental paradigm to show that the same phonetic contrast can be associated with seemingly disparate dimensions such as roughness, taste and gender. Together, these studies suggest that pluripotentiality is a core feature of sound symbolism.

### (c) Is question intonation linked to body size?

As noted above, a final dimension of the frequency code proposal relates to question intonation. Ohala [14] suggested that the cross-linguistic tendency for questions as opposed to statements to be associated with rising contours could also be related to a biological motivation grounded in body size [46], specifically, the idea that vocal markers of submissiveness and politeness may explain why questions are rising, as opposed to falling. There are two questions we need to ask here: (i) how strong is the evidence for the cross-linguistic tendency? and (ii), if the cross-linguistic generalization exists, does the frequency code actually motivate it?

Indeed, it is reported for many diverse languages that questions are typically marked with rising contours, including Cantonese [140], German [141], Dutch [142], Spanish [143], Bengali [144], Georgian [145], Mongolian [146], Lebanese and Egyptian Arabic [147], and many others. There are also studies that directly compare the intonational meaning across languages within the same paradigm, such as Gussenhoven & Chen [148], who found that Dutch, Hungarian and Mandarin Chinese listeners agree with each other in perceiving rising contours as indicators of questions. There are, however, also several languages that have the opposite pattern (questions = falling contour), such as reported for several Bantu languages by Rialland [149].^2^ Using a high rising contour for statements is also widely attested in several varieties of English, sometimes discussed as ‘uptalk’ [3,152–154]. However, the precise intonation contour for ‘uptalk’ may differ from rising contours in questions [155]. This points to an issue of the frequency code proposal, which is that the concept of a ‘rising contour’ (and conversely, that of a ‘falling contour’) is so broad that it subsumes a number of quite distinct intonation contours. In a similar fashion, Ladd [151, p. 1382] discusses universals in intonation and criticizes the fact that accounts such as the frequency code are ‘effectively unfalsifiable’. How, for example, should question contours that have sharp final rises followed by sharp falls be treated? Does the sharp rise count as positive evidence for the frequency code even though it is immediately followed by a fall? It is furthermore worth noting that although the rising = question/falling = statement pattern has been observed in individual studies of the intonational systems of specific languages, there has been no work that actually provides a formal test for this idea with a large sample of languages controlling for genealogical and areal dependencies, as is standard in modern statistical typology [121,156].

Even if, for the time being, we accept that rising question intonation is a cross-linguistic tendency, the explanatory connection between body size and sentence type (question versus statement) is exceedingly indirect, requiring multiple linking assumptions. The idea that questions relate to some notion of ‘informational submissiveness' or in Ohala's terms, ‘desire for the goodwill of the receiver’ [9,10,12, p. 343] may appear plausible, but the explanation itself is not directly supported by any data. This is especially true in the light of our discussion of pluripotentiality: the fact that pitch serves multiple functions means that alternative explanations of the universal tendencies are possible and have indeed been proposed (e.g. [22]). This was also pointed out by Ladd [151], who noted that it is hard to establish with direct evidence whether a universalist explanation is correct. In the absence of direct empirical evidence for the frequency code, it is only one plausible explanation among others. It is important to note that the available evidence *that* there are universal tendencies in question intonation underspecifies any one explanatory account without the incorporation of additional evidence. ‘Biological codes’, such as the frequency code, are an additional interpretational layer that can help researchers make sense of empirical data and synthesize different communicative facts across studies, but it is hard to find evidence that directly supports a specific biology-based explanation, such as the frequency code, above and beyond other explanations, especially given the multidimensional nature of pitch.

## 5. Conclusion

We began by reviewing the frequency code hypothesis according to which a number of communicative interpretations of pitch are grounded in associated differences in body size. As reviewed above, aspects of the proposal, such as the association between low *F*0 and dominance or the presence of universal tendencies in size sound symbolism have been empirically confirmed. For other aspects of the frequency code, the evidence is mixed. In particular, when it comes to the culturally and socially variegated phenomenon of politeness, predictions have not been borne out consistently across studies and across languages. Our meta-analysis showed that there is by now a sizeable portion of studies from a small but genealogically diverse sample of languages in which *low* pitch is associated with a deferential form of polite speech. We have suggested that prosodic mitigation is a plausible explanation of this observation, especially given that this account is consistent with a number of other facts (e.g. other phonetic cues that suggest mitigated speech, reduction in gesture, reduced facial expressivity). Moreover, we have suggested that some of the interpretations of high pitch (such as animated speech and other intense emotions) are incompatible with particular forms of politeness.

A guiding concept for our discussion was the idea of ‘pluripotentiality’: any linguistic form can serve multiple different functions depending on a large number of contextual factors. This pluripotentiality was already implicitly referenced in Ohala's proposal, but it is now backed up by substantial empirical evidence. The pluripotentiality is apparent when looking at the meaning of pitch across different studies, or based on tasks which directly show that contextual manipulations influence the interpretation of sound symbolic stimuli [139], or the politeness-related interpretation of pitch [83]. The fact that pitch has multiple different interpretations also means that we cannot easily ground proposed intonation universals in the frequency code without additional evidence. We thus think that while the frequency code has been useful in some domains of inquiry, its application to communicative phenomena less directly connected to size is considerably more tenuous. Given that the frequency code is by its very nature an umbrella proposal, linking seemingly disparate facts, it is important to ask the questions whether all empirical phenomena are actually linked to particular biological explanations, and whether this link is direct, or mediated by additional cultural factors.

## Footnotes

1 Clearly, Ohala's explanation only pertains to a certain type of question. Questions can also be coming from a point of authority and dominance, such as in a court hearing or interrogation. Questions can also have a prosocial function, such as when offering something (e.g. *Do you want some coffee?*). In these situations, the question asker is ‘self-sufficient’ and not ‘appealing to the addressee for help’. The fact that questions themselves perform a whole range of different communicative functions is not discussed by Ohala.

2 Gussenhoven [150] proposes that the seemingly exceptional pattern of many African languages noted by Rialland can be explained by assuming that breathiness is the primary feature, with low voice pitch following from this. He proposes the ‘sirenic code’ that involves associated meanings stemming from the fact that breathiness is associated with femininity. However, if exceptions to the frequency code can be explained away by invoking other biological codes, it is not clear how the idea that the frequency code underlies question intonation could be falsified (see [151]).

## Data accessibility

All data and code are available from the following repository: https://osf.io/amw7u/.

## References

[1] Büring D. 2007 Semantics, intonation and information structure. In The Oxford handbook of linguistic interfaces, pp. 445-474. Oxford, UK: Oxford University Press.

[2] Pierrehumbert J, Hirschberg JB. 1990 The meaning of intonational contours in the interpretation of discourse. In Intentions in communication (eds PR Cohen, J Morgan, M Pollack), pp. 271-311. Cambridge, MA: MIT Press.

[3] Cruttenden A. 1997 Intonation. Cambridge, UK: Cambridge University Press.

[4] Pierrehumbert J. 1980 The phonology and phonetics of English intonation. PhD thesis, Massachusetts Institute of Technology, Cambridge, MA, USA.

[5] Cheng C-C. 2011 A synchronic phonology of Mandarin Chinese. Berlin, Germany: Walter de Gruyter.

[6] Hyman LM. 2001 Tone systems. In Language typology and language universals (eds M Haspelmath, E König, W Osterreicher, W Raible), pp. 1367-1380. Berlin, Germany: Walter de Gruyter.

[7] Banse R, Scherer KR. 1996 Acoustic profiles in vocal emotion expression. J. Pers. Soc. Psychol. **70**, 614. (10.1037/0022-3514.70.3.614) 8851745

[8] Scherer KR. 1986 Vocal affect expression: a review and a model for future research. Psychol. Bull. **99**, 143. (10.1037/0033-2909.99.2.143) 3515381

[9] Hodges-Simeon CR, Gaulin SJ, Puts DA. 2010 Different vocal parameters predict perceptions of dominance and attractiveness. Hum. Nat. **21**, 406-427. (10.1007/s12110-010-9101-5) 21212816 PMC2995855

[10] Puts DA, Gaulin SJ, Verdolini K. 2006 Dominance and the evolution of sexual dimorphism in human voice pitch. Evol. Hum. Behav. **27**, 283-296. (10.1016/j.evolhumbehav.2005.11.003)

[11] Sell A, Bryant GA, Cosmides L, Tooby J, Sznycer D, von Rueden C, Krauss A, Gurven M. 2010 Adaptations in humans for assessing physical strength from the voice. Proc. R. Soc. B **277**, 3509-3518. (10.1098/rspb.2010.0769) PMC2982226 20554544

[12] Wolff SE, Puts DA. 2010 Vocal masculinity is a robust dominance signal in men. Behav. Ecol. Sociobiol. **64**, 1673-1683. (10.1007/s00265-010-0981-5)

[13] Uldall E. 1960 Attitudinal meanings conveyed by intonation contours. Lang. Speech **3**, 223-234. (10.1177/002383096000300403)

[14] Ohala JJ. 1994 The frequency code underlies the sound-symbolic use of voice pitch. In Sound symbolism (eds L Hinton, J Nichols, JJ Ohala), pp. 325-347. Cambridge, UK: Cambridge University Press.

[15] Bowling DL, Garcia M, Dunn JC, Ruprecht R, Stewart A, Frommolt K-H, Fitch WT. 2017 Body size and vocalization in primates and carnivores. Sci. Rep. **7**, 1-11. (10.1038/srep41070) 28117380 PMC5259760

[16] Fitch WT. 1997 Vocal tract length and formant frequency dispersion correlate with body size in rhesus macaques. J. Acoust. Soc. Am. **102**, 1213-1222. (10.1121/1.421048) 9265764

[17] Gingras B, Boeckle M, Herbst CT, Fitch WT. 2013 Call acoustics reflect body size across four clades of anurans. J. Zool. **289**, 143-150. (10.1111/j.1469-7998.2012.00973.x)

[18] Dunn JC, Halenar LB, Davies TG, Cristobal-Azkarate J, Reby D, Sykes D, Dengg S, Fitch WT, Knapp LA. 2015 Evolutionary trade-off between vocal tract and testes dimensions in howler monkeys. Curr. Biol. **25**, 2839-2844. (10.1016/j.cub.2015.09.029) 26592343 PMC4635310

[19] Pietraszewski D, Wertz AE, Bryant GA, Wynn K. 2017 Three-month-old human infants use vocal cues of body size. Proc. R. Soc. B **284**, 20170656. (10.1098/rspb.2017.0656) PMC5474077 28592674

[20] Rendall D, Nemeth C. 2007 Lifting the curtain on the Wizard of Oz: biased voice-based impressions of speaker size. J. Exp. Psychol. Hum. Percept. Perform. **33**, 1208-1219. (10.1037/0096-1523.33.5.1208) 17924818

[21] Bolinger D. 1978 Intonation across languages. In Universals of human language, vol 2: phonology (eds Joseph H. Greenberg, CA Ferguson, EA Moravcsik), pp. 471-524. Palo Alto, CA: Stanford University Press.

[22] Cruttenden A. 1981 Falls and rises: meanings and universals. J. Linguist. **17**, 77-91. (10.1017/S0022226700006782)

[23] Gussenhoven C. 2004 The phonology of tone and intonation. Cambridge, UK: Cambridge University Press.

[24] Taylor AM, Reby D. 2010 The contribution of source–filter theory to mammal vocal communication research. J. Zool. **280**, 221-236. (10.1111/j.1469-7998.2009.00661.x)

[25] Morton ES. 1977 On the occurrence and significance of motivation-structural rules in some bird and mammal sounds. Am. Nat. **111**, 855-869. (10.1086/283219)

[26] Wallschläger D. 1980 Correlation of song frequency and body weight in passerine birds. Experientia **36**, 412. (10.1007/BF01975119)

[27] Charlton BD, Reby D. 2016 The evolution of acoustic size exaggeration in terrestrial mammals. Nat. Commun. **7**, 1-8. (10.1038/ncomms12739) PMC5025854 27598835

[28] Reby D, McComb K, Cargnelutti B, Darwin C, Fitch WT, Clutton-Brock T. 2005 Red deer stags use formants as assessment cues during intrasexual agonistic interactions. Proc. R. Soc. B **272**, 941-947. (10.1098/rspb.2004.2954) PMC1564087 16024350

[29] Vannoni E, McElligott AG. 2008 Low frequency groans indicate larger and more dominant fallow deer (*Dama dama*) males. PLoS ONE **3**, e3113. (10.1371/journal.pone.0003113) 18769619 PMC2518835

[30] Reber SA, Janisch J, Torregrosa K, Darlington J, Vliet KA, Fitch WT. 2017 Formants provide honest acoustic cues to body size in American alligators. Sci. Rep. **7**, 1-11. (10.1006/anbe.1998.0756) 28500350 PMC5431764

[31] Pfefferle D, West PM, Grinnell J, Packer C, Fischer J. 2007 Do acoustic features of lion, *Panthera leo*, roars reflect sex and male condition? J. Acoust. Soc. Am. **121**, 3947-3953. (10.1121/1.2722507) 17552741

[32] Vannoni E, McElligott AG. 2007 Individual acoustic variation in fallow deer (*Dama dama*) common and harsh groans: a source-filter theory perspective. Ethology **113**, 223-234. (10.1111/j.1439-0310.2006.01323.x)

[33] Klatt DH, Klatt LC. 1990 Analysis, synthesis, and perception of voice quality variations among female and male talkers. J. Acoust. Soc. Am. **87**, 820-857. (10.1121/1.398894) 2137837

[34] Biemans M. 1998 The effect of biological gender (sex) and social gender (gender identity) on three pitch measures. Linguist. Neth. **15**, 41-52. (10.1075/avt.15.06bie)

[35] Podesva RJ. 2011 Salience and the social meaning of declarative contours: three case studies of gay professionals. J. Engl. Linguist. **39**, 233-264. (10.1177/0075424211405161)

[36] Ohara Y. 2001 Finding one's voice in Japanese: a study of the pitch levels of L2 users. In Multilingualism, second language learning, and gender (eds A Pavlenko, A Brackledge, I Piller, M Teutsch-Dwyer), pp. 231-254. New York, NY: Mouton de Gruyter.

[37] Nagels L, Gaudrain E, Vickers D, Hendriks P, Başkent D. 2020 Development of voice perception is dissociated across gender cues in school-age children. Sci. Rep. **10**, 5074. (10.1038/s41598-020-61732-6) 32193411 PMC7081243

[38] Green EJ, Barber PJ. 1981 An auditory Stroop effect with judgments of speaker gender. Percept. Psychophys. **30**, 459-466. (10.3758/BF03204842) 7329763

[39] Most SB, Sorber AV, Cunningham JG. 2007 Auditory Stroop reveals implicit gender associations in adults and children. J. Exp. Soc. Psychol. **43**, 287-294. (10.1016/j.jesp.2006.02.002)

[40] Gilbert HR, Robb MP. 1996 Vocal fundamental frequency characteristics of infant hunger cries: birth to 12 months. Int. J. Pediatr. Otorhinolaryngol. **34**, 237-243. (10.1016/0165-5876(95)01273-7) 8839074

[41] Murry T, Amundson P, Hollien H. 1977 Acoustical characteristics of infant cries: fundamental frequency. J. Child Lang. **4**, 321-328. (10.1017/S0305000900001719)

[42] Evans S, Neave N, Wakelin D. 2006 Relationships between vocal characteristics and body size and shape in human males: an evolutionary explanation for a deep male voice. Biol. Psychol. **72**, 160-163. (10.1016/j.biopsycho.2005.09.003) 16280195

[43] Rendall D, Kollias S, Ney C, Lloyd P. 2005 Pitch (F0) and formant profiles of human vowels and vowel-like baboon grunts: the role of vocalizer body size and voice-acoustic allometry. J. Acoust. Soc. Am. **117**, 944-955. (10.1121/1.1848011) 15759713

[44] van Dommelen WA, Moxness BH. 1995 Acoustic parameters in speaker height and weight identification: sex-specific behaviour. Lang. Speech **38**, 267-287. (10.1177/002383099503800304) 8816083

[45] Pisanski K, Mora EC, Pisanski A, Reby D, Sorokowski P, Frackowiak T, Feinberg DR. 2016 Volitional exaggeration of body size through fundamental and formant frequency modulation in humans. Sci. Rep. **6**, 1-8. (10.1038/srep34389) 27687571 PMC5043380

[46] Gussenhoven C. 2002 Intonation and interpretation: phonetics and phonology. In Speech Prosody 2002, Int. Conf., 11–13 April 2002, Aix-en-Provence, France, pp. 47-57. Baixas, France: ISCA.

[47] Sell A, Cosmides L, Tooby J, Sznycer D, von Rueden C, Gurven M. 2009 Human adaptations for the visual assessment of strength and fighting ability from the body and face. Proc. R. Soc. B **276**, 575-584. (10.1098/rspb.2008.1177) PMC2664345 18945661

[48] Dabbs JM, Mallinger A. 1999 High testosterone levels predict low voice pitch among men. Pers. Individ. Differ. **27**, 801-804. (10.1016/S0191-8869(98)00272-4)

[49] Evans S, Neave N, Wakelin D, Hamilton C. 2008 The relationship between testosterone and vocal frequencies in human males. Physiol. Behav. **93**, 783-788. (10.1016/j.physbeh.2007.11.033) 18155094

[50] Meuser W, Nieschlag E. 1977 Sexualhormone und Stimmlage des Mannes. DMW-Deutsche Medizinische Wochenschrift **102**, 261-264. (10.1055/s-0028-1104875) 138584

[51] Watts RJ. 2003 Politeness. Cambridge, UK: Cambridge University Press.

[52] Meier AJ. 1995 Defining politeness: universality in appropriateness. Lang. Sci. **17**, 345-356. (10.1016/0388-0001(95)00019-4)

[53] Culpeper J. 2011 Impoliteness: using language to cause offence. Cambridge, UK: Cambridge University Press.

[54] Brown P, Levinson SC. 1987 Politeness: some universals in language usage. Cambridge, UK: Cambridge University Press.

[55] Kádár DZ, Haugh M. 2013 Understanding politeness. Cambridge, UK: Cambridge University Press.

[56] Loveday L. 1981 Pitch, politeness and sexual role: an exploratory investigation into the pitch correlates of English and Japanese politeness formulae. Lang. Speech **24**, 71-89. (10.1177/002383098102400105)

[57] Pike KL. 1945 The intonation of American English. Ann Arbor, MI: University of Michigan Press.

[58] Winter B, Perlman M. 2021 Size sound symbolism in the English lexicon. Glossa: J. Gen. Linguist. **6**, 1-13. (10.5334/gjgl.1646)

[59] Blasi DE, Wichmann S, Hammarström H, Stadler PF, Christiansen MH. 2016 Sound–meaning association biases evidenced across thousands of languages. Proc. Natl Acad. Sci. USA **113**, 10 818-10 823. (10.1073/pnas.1605782113) 27621455 PMC5047153

[60] Fitch WT. 1994 Vocal tract length perception and the evolution of language. BA thesis, Brown University, Providence, RI, USA.

[61] Jespersen O. 1922 Language: its nature and development. New York, NY: Henry Holt and Company.

[62] Johansson N, Anikin A, Carling G, Holmer A. 2019 The typology of sound symbolism: defining macro-concepts via their semantic and phonetic features. Linguist. Typol. 24, 253-310.

[63] Shih S, Rudin D. 2020 On sound symbolism in baseball player names. Names 69, 20-35. (10.5195/names.2021.2245)

[64] Auracher J. 2017 Sound iconicity of abstract concepts: place of articulation is implicitly associated with abstract concepts of size and social dominance. PLoS ONE **12**, e0187196. (10.1371/journal.pone.0187196) 29091943 PMC5665516

[65] Klink RR. 2000 Creating brand names with meaning: the use of sound symbolism. Market. Lett. **11**, 5-20. (10.1023/A:1008184423824)

[66] Knoeferle K, Li J, Maggioni E, Spence C. 2017 What drives sound symbolism? Different acoustic cues underlie sound-size and sound-shape mappings. Sci. Rep. **7**, 5562. (10.1038/s41598-017-05965-y) 28717151 PMC5514121

[67] Newman SS. 1933 Further experiments in phonetic symbolism. Am. J. Psychol. **45**, 53-75. (10.2307/1414186)

[68] Sapir E. 1929 A study in phonetic symbolism. J. Exp. Psychol. **12**, 225-239. (10.1037/h0070931)

[69] Hoshi H, Kwon N, Akita K, Auracher J 2019 Semantic associations dominate over perceptual associations in vowel–size iconicity. i-Perception **10**, 2041669519861981. (10.1177/2041669519861981) 31321019 PMC6628535

[70] Huang Y-H, Pratoomraj S, Johnson RC. 1969 Universal magnitude symbolism. J. Verb. Learn. Verb. Behav. **8**, 155-156. (10.1016/S0022-5371(69)80028-9)

[71] Kawahara S, Kumagai G. 2019 Expressing evolution in Pokémon names: experimental explorations. J. Jpn Linguist. **35**, 3-38. (10.1515/jjl-2019-2002)

[72] LaPolla RJ. 1994 An experimental investigation into phonetic symbolism as it relates to Mandarin Chinese. In Sound symbolism (eds L Hinton, J Nichols, JJ Ohala), pp. 130-147. Cambridge, UK: Cambridge University Press.

[73] Markel NN, Hamp EP. 1960 Connotative meanings of certain phoneme sequences. Stud. Linguist. **15**, 47-61.

[74] Tarte RD. 1974 Phonetic symbolism in adult native speakers of Czech. Lang. Speech **17**, 87-94. (10.1177/002383097401700109) 5566128

[75] Westermann DH. 1927 Laut, Ton und Sinn in westafrikanischen Sudansprachen. Festschrift Meinhof **315**, 315-326.

[76] Childs GT. 1994 African ideophones. In Sound Symbolism (eds L Hinton, J Nichols, JJ Ohala), pp. 178–206. Cambridge, UK: Cambridge University Press.

[77] Wescott RW. 1973 Tonal icons in Bini. Stud. Afr. Linguist. **4**, 197.

[78] Bolinger D. 1964 Intonation as a universal. In Proc. of the Ninth International Congress of Linguistics (ed. HG Lunt), pp. 833-844. The Hague, The Netherlands: Mouton de Gruyter.

[79] Hermann E. 1942 Probleme der Frage. Göttingen, Germany: Vandenhoeck & Ruprecht.

[80] Brown L. 2015 Honorifics and politeness. In The handbook of Korean linguistics (eds L Brown, J Yeon), pp. 303-319. Malden, MA: Wiley Blackwell.

[81] Byon AS. 2006 The role of linguistic indirectness and honorifics in achieving linguistic politeness in Korean requests. J. Polit. Res. Lang. Behav. Cult. **2**, 247-276. (10.1515/PR.2006.013)

[82] Yoon K-J. 2004 Not just words: Korean social models and the use of honorifics. Intercult. Pragmat. **1**, 189-210. (10.1515/iprg.2004.1.2.189)

[83] Nadeu M, Prieto P. 2011 Pitch range, gestural information, and perceived politeness in Catalan. J. Pragmat. **43**, 841-854. (10.1016/j.pragma.2010.09.015)

[84] McKinnon S, Prieto Vives P. 2014 The role of prosody and gesture in the perception of mock impoliteness. J. Polit. Res. **10**, 185-219. (10.1515/pr-2014-0009)

[85] Culpeper J. 2011 It's not what you said, it's how you said it!: prosody and impoliteness. In Discursive approaches to politeness (ed. Linguistics Politeness Research Group), pp. 57-83. Berlin, Germany: Mouton de Gruyter.

[86] Culpeper J, Bousfield D, Wichmann A. 2003 Impoliteness revisited: with special reference to dynamic and prosodic aspects. J. Pragmat. **35**, 1545-1579. (10.1016/S0378-2166(02)00118-2)

[87] Winter B, Grawunder S. 2012 The phonetic profile of Korean formal and informal speech registers. J. Phon. **40**, 808-815. (10.1016/j.wocn.2012.08.006)

[88] Brown L, Winter B. 2019 Multimodal indexicality in Korean: ‘doing deference’ and ‘performing intimacy’ through nonverbal behavior. J. Polit. Res. **15**, 25-54. (10.1515/pr-2016-0042)

[89] Brown L, Prieto P. 2017 (Im) politeness: prosody and gesture. In The Palgrave handbook of linguistic (im) politeness (eds J Culpeper, M Haugh, DZ Kádár), pp. 357-379. Berlin, Germany: Springer.

[90] Hübscher I, Garufi M, Prieto P. 2019 The development of polite stance in preschoolers: how prosody, gesture, and body cues pave the way. J. Child Lang. **46**, 825-862. (10.1017/S0305000919000126) 31099328

[91] Tsuji A. 2004 The case study of high pitch register in English and in Japanese: does high pitch register relate to politeness. Seijo Engl. Monogr. **37**, 227-260.

[92] Ofuka E, McKeown JD, Waterman MG, Roach PJ. 2000 Prosodic cues for rated politeness in Japanese speech. Speech Commun. **32**, 199-217. (10.1016/S0167-6393(00)00009-1)

[93] Caballero JA, Vergis N, Jiang X, Pell MD. 2018 The sound of im/politeness. Speech Commun. **102**, 39-53. (10.1016/j.specom.2018.06.004)

[94] Orozco L. 2008. *Peticiones corteses y factores prosódicos*. In *Fonología instrumental* (eds E Herrera Zendejas, M Butragueno), pp. 335-355. Mexico City, Mexico: El Colegio de México.

[95] Goodwin MH, Goodwin C, Yaeger-Dror M. 2002 Multi-modality in girls' game disputes. J. Pragmat. **34**, 1621-1649. (10.1016/S0378-2166(02)00078-4)

[96] Chen A, Gussenhoven C, Rietveld T. 2004 Language-specificity in the perception of paralinguistic intonational meaning. Lang. Speech **47**, 311-349. (10.1177/00238309040470040101) 16038447

[97] Shin S. 2005 Grammaticalization of politeness: a contrastive study of German, English and Korean. Berkeley, CA: University of California.

[98] Stadler SA. 2006 Multimodal (im) politeness: the verbal, prosodic and non-verbal realization of disagreement in German and New Zealand English. PhD thesis, University of Auckland, Aukland, New Zealand.

[99] Devís Herraiz E, Cantero Serena FJ. 2014 The intonation of mitigating politeness in Catalan. J. Polit. Res. **10**, 127-149. (10.1515/pr-2014-0006)

[100] Grawunder S, Oertel M, Schwarze C. 2014 Politeness, culture, and speaking task—paralinguistic prosodic behavior of speakers from Austria and Germany. In Conf. Speech prosody, 20–23 May 2014, Dublin, Republic of Ireland, pp. 159–163. Baixas, France: ISCA.

[101] Hübscher I, Borràs-Comes J, Prieto P. 2017 Prosodic mitigation characterizes Catalan formal speech: the frequency code reassessed. J. Phon. **65**, 145-159. (10.1016/j.wocn.2017.07.001)

[102] Chikulaeva A, D'Imperio M. 2018 The expression of politeness and pitch height in Russian imperatives. In Conf. Speech Prosody, 13–16 June 2018, Poznań, Poland, pp. 438–442. Baixas, France: ISCA.

[103] Idemaru K, Winter B, Brown L. 2019 Cross-cultural multimodal politeness: the phonetics of Japanese deferential speech in comparison to Korean. Intercult. Pragmat. **16**, 517-555. (10.1515/ip-2019-0027)

[104] Sherr-Ziarko E. 2019 Prosodic properties of formality in conversational Japanese. J. Int. Phon. Assoc. **49**, 331-352. (10.1017/S0025100318000117)

[105] Idemaru K, Winter B, Brown L, Oh GE. 2020 Loudness trumps pitch in politeness judgments: evidence from Korean deferential speech. Lang. Speech **63**, 123-148. (10.1177/0023830918824344) 30732514

[106] Gucek R, Le Gac D. 2019 Prosodic manifestations of politeness in Porteño Spanish wh-interrogatives: terminal contours, f0 mitigation and syllable durations. In Proc. of the 19th Int. Congress of Phonetic Sciences, 5–9 August 2019, Melbourne, Australia, pp. 3388-3392. Canberra City, Australia: Australasian Speech Science and Technology Association Inc.

[107] Oh E, Cui M. 2020 The acquisition of acoustic correlates of politeness by native Chinese speakers. Linguist. Res. **37**, 113-134. (10.17250/khisli.37.202009.005)

[108] Brown L, Winter B, Idemaru K, Grawunder S. 2014 Phonetics and politeness: perceiving Korean honorific and non-honorific speech through phonetic cues. J. Pragmat. **66**, 45-60. (10.1016/j.pragma.2014.02.011)

[109] Ambady N, Koo J, Lee F, Rosenthal R. 1996 More than words: linguistic and nonlinguistic politeness in two cultures. J. Pers. Soc. Psychol. **70**, 996. (10.1037/0022-3514.70.5.996)

[110] Ito M. 2004 Politeness and voice quality-the alternative method to measure aspiration noise. In Speech Prosody 2004, Int. Conf. 23–26 March 2004, Nara, Japan. Baixas, France: ISCA.

[111] Lin H-Y, Tse K-PJ, Fon J. 2006 An acoustic study on the paralinguistic prosody in the politeness talk in Taiwan Mandarin. In ITRW on experimental linguistics. (ed. A Botinis), pp. 173–176. Baixas, France: ISCA.

[112] Ogiermann E. 2018 Discourse completion tasks. In Methods in pragmatics (eds A Jucker, K Schneider, W Bublitz), pp. 229-255. Berlin, Germany: Mouton de Gruyter.

[113] Ide S. 1989 Formal forms and discernment: two neglected aspects of universals of linguistic politeness. Multilin. J. Cross-cult. Interlang. Commun. **8**, 223-248. (10.1515/mult.1989.8.2-3.223)

[114] Leech GN. 2014 The pragmatics of politeness. Oxford, UK: Oxford University Press.

[115] Brown L. 2013 ‘Mind your own esteemed business': sarcastic honorifics use and impoliteness in Korean TV dramas. J. Polit. Res. **9**, 159-186. (10.1515/pr-2013-0008)

[116] Boersma P, Weenink D. 2018 Praat: doing phonetics by computer. Version 6.0. 37. Retrieved 3 February 2018. See https://www.fon.hum.uva.nl/praat/.

[117] Yarkoni T. In press. The generalizability crisis. Behav. Brain Sci. 1-37. (10.1017/S0140525X20001685) PMC10681374 33342451

[118] R Core Team. 2019 R: a language and environment for statistical computing. Vienna, Austria: R Foundation for Statistical Computing.

[119] Wickham H et al. 2019 Welcome to the Tidyverse. J. Open Source Softw. **4**, 1686. (10.21105/joss.01686)

[120] Bürkner P-C. 2017 brms: an R package for Bayesian multilevel models using Stan. J. Stat. Softw. **80**, 1-28. (10.18637/jss.v080.i01)

[121] Jaeger TF, Graff P, Croft W, Pontillo D. 2011 Mixed effect models for genetic and areal dependencies in linguistic typology. Linguist. Typol. **15**, 281-319. (10.1515/lity.2011.021)

[122] Cysouw M. 2010 Dealing with diversity: towards an explanation of NP-internal word order frequencies. Linguist. Typol. **14**, 253-286. (10.1515/lity.2010.010)

[123] Frick RW. 1986 The prosodic expression of anger: differentiating threat and frustration. Aggress. Behav. **12**, 121-128. (10.1002/1098-2337(1986)12:2<121::AID-AB2480120206>3.0.CO;2-F)

[124] Hübscher I, Sánchez-Conde C, Borràs-Comes J, Vincze L, Prieto P. Submitted. Multimodal mitigation: how facial and body cues index politeness in Catalan requests. J. Polit. Res.

[125] Werner H, Kaplan B. 1963 Symbol formation. New York, NY: John Wiley & Sons.

[126] Sidhu DM, Pexman PM. 2018 Lonely sensational icons: semantic neighbourhood density, sensory experience and iconicity. Lang. Cognit. Neurosci. **33**, 25-31. (10.1080/23273798.2017.1358379)

[127] Ertel S. 1965 Der Lautcharakter künstlicher Lautgebilde. Psychologische Forschung **28**, 491-518. (10.1007/BF00421741) 4158322

[128] O'Boyle MW, Tarte RD. 1980 Implications for phonetic symbolism: the relationship between pure tones and geometric figures. J. Psycholinguist. Res. **9**, 535-544. (10.1007/BF01068115) 6162950

[129] Marks LE. 1974 On associations of light and sound: the mediation of brightness, pitch, and loudness. Am. J. Psychol. 87, 173-188. (10.2307/1422011) 4451203

[130] Marks LE. 1989 On cross-modal similarity: the perceptual structure of pitch, loudness, and brightness. J. Exp. Psychol. Hum. Percept. Perform. **15**, 586. (10.1037/0096-1523.15.3.586) 2527964

[131] Marks LE. 1975 On colored-hearing synesthesia: cross-modal translations of sensory dimensions. Psychol. Bull. **82**, 303. (10.1037/0033-2909.82.3.303) 1096209

[132] Marks LE. 1982 Bright sneezes and dark coughs, loud sunlight and soft moonlight. J. Exp. Psychol. Hum. Percept. Perform. **8**, 177. (10.1037//0096-1523.8.2.177) 6461716

[133] Lockwood G, Dingemanse M. 2015 Iconicity in the lab: a review of behavioral, developmental, and neuroimaging research into sound-symbolism. Front. Psychol. **6**, 1246. (10.3389/fpsyg.2015.01246) 26379581 PMC4547014

[134] French PL. 1977 Toward an explanation of phonetic symbolism. Word **28**, 305-322. (10.1080/00437956.1977.11435647)

[135] Miall DS. 2001 Sounds of contrast: an empirical approach to phonemic iconicity. Poetics **29**, 55-70. (10.1016/S0304-422X(00)00025-5)

[136] Bredin H. 1996 Onomatopoeia as a figure and a linguistic principle. New Lit. Hist. **27**, 555-569. (10.1353/nlh.1996.0031)

[137] Hrushovski B. 1980 The meaning of sound patterns in poetry: an interaction theory. Poetics Today **2**, 39-56. (10.2307/1772351)

[138] Tsur R. 2012 Playing by ear and the tip of the tongue: precategorical information in poetry. Amsterdam, The Netherlands: John Benjamins.

[139] Winter B, Pérez-Sobrino P, Brown L. 2019 The sound of soft alcohol: crossmodal associations between interjections and liquor. PLoS ONE **14**, e0220449. (10.1371/journal.pone.0220449) 31393912 PMC6687133

[140] Ma JK, Ciocca V, Whitehill TL. 2006 Effect of intonation on Cantonese lexical tones. J. Acoust. Soc. Am. **120**, 3978-3987. (10.1121/1.2363927) 17225424

[141] Grice M, Baumann S, Benzmüller R. 2006 Autosegmental-metrical phonology. In Prosodic typology: the phonology of intonation and phrasing (ed. S-A Jun), pp. 55-83. Cambridge, UK: Cambridge University Press.

[142] Haan J, Van Heuven VJ. 1997 An anatomy of Dutch question intonation. In Linguistics in the Netherlands (eds JA Coerts, H de Hoop), pp. 97-108. Amsterdam, The Netherlands: John Benjamins.

[143] Hualde JI, Prieto P. 2015 Intonational variation in Spanish: European and American varieties. In Intonation in romance (eds S Frota, P Prieto), pp. 350-391. Oxford, UK: Oxford University Press.

[144] Kahn SD. 2015 The intonational phonology of Bangladeshi Standard Bengali. In Prosodic typology: the phonology of intonation and phrasing 2 (ed. S-A Jun), pp. 81-117. Oxford, UK: Oxford University Press.

[145] Vicenik C, Jun S-A. 2015 Autosegmental-metrical analysis of Georgian intonation. In Prosodic typology: the phonology of intonation and phrasing 2 (ed. S-A Jun), pp. 154-186. Oxford, UK: Oxford University Press.

[146] Karlsson A. 2015 The intonational phonology of Mongolian. In *Prosodic typology: the phonology of intonation and phrasing 2* (ed. S-A Jun), pp. 187-215. Oxford, UK: Oxford University Press.

[147] Chahal D, Hellmuth S. 2014 The intonation of Lebanese and Egyptian Arabic. In Prosodic typology: the phonology of intonation and phrasing 2 (ed. S-A Jun), pp. 365-405. Oxford, UK: Oxford University Press.

[148] Gussenhoven C, Chen A. 2000 Universal and language-specific effects in the perception of question intonation. In 6th Int. Conf. on Spoken Language Processing (ICSLP 2000), 16–20 October 2000, Beijing, China. pp. 91-94. Baixas, France: ISCA.

[149] Rialland A. 2009 The African lax question prosody: its realisation and geographical distribution. Lingua **119**, 928-949. (10.1016/j.lingua.2007.09.014)

[150] Gussenhoven C. 2016 Foundations of intonational meaning: anatomical and physiological factors. Topics Cognit. Sci. **8**, 425-434. (10.1111/tops.12197) 27016315

[151] Ladd R. 2001 Intonation. In Language typology and language universals (eds M Haspelmath, E König, W Osterreicher, W Raible), pp. 1380-1390. Berlin, Germany: Walter de Gruyter.

[152] Fletcher J, Grabe E, Warren P. 2005 Intonational variation in four dialects of English: the high rising tune. In Prosodic typology, the phonology of intonation and phrasing 2 (ed. S–A Jun), pp. 390-409. Oxford, UK: Oxford University Press.

[153] Guy G, Horvath B, Vonwiller J, Daisley E, Rogers I. 1986 An intonational change in progress in Australian English. Lang. Soc. **15**, 23-51. (10.1017/S0047404500011635)

[154] Warren P. 2016 Uptalk: the phenomenon of rising intonation. Cambridge, UK: Cambridge University Press.

[155] Warren P, Fletcher J. 2016 Phonetic differences between uptalk and question rises in two Antipodean English varieties. In Conf. Speech Prosody, 31 May–3 June 2016, Boston, USA, pp. 148–152. Baixas, France: ISCA.

[156] Bickel B. 2015 Distributional typology: statistical inquiries into the dynamics of linguistic diversity. In *The Oxford handbook of linguistic analysis* (eds B Heine, H Narrog), pp. 901–924. Oxford, UK: Oxford University Press.

